# Every coin has two sides: ChatGPT poses a potential threat to Nursing Students' Education

**DOI:** 10.3389/fmed.2024.1415067

**Published:** 2024-07-24

**Authors:** Amirhossein Sharifi Kelarijani, Ali Safdari, Mohamad Golitaleb

**Affiliations:** ^1^Student Research Committee, Hamadan University of Medical Sciences, Hamedan, Iran; ^2^Department of Critical Care Nursing, School of Nursing and Midwifery, Tehran University of Medical Sciences, Tehran, Iran; ^3^Department of Nursing, School of Nursing, Arak University of Medical Sciences, Arak, Iran

**Keywords:** nursing - education, ChatGPT, nursing, health profession education, nursing student

One recent example of artificial intelligence is the generative pre-trained transformer (ChatGPT) perceived as a conversational chatbot launched by the artificial intelligence company OpenAI^®^ in 2022 and quickly gained widespread popularity (1). Recently, we have seen more research focusing on developing artificial intelligence technologies in nursing. However, most existing studies focus on the capabilities and functionalities of this robot, while due to the novelty of this technology application, there are extensive unknown dimensions associated with it. Therefore, identifying all dimensions of it requires time. These sensitivities are particularly more relevant in the education of nursing students. As nursing care continues to evolve, nursing education must also evolve. Today, the development of artificial intelligence technologies and technological advancements herald significant changes in the future of nursing (2).

Nurses constitute a vital part of the healthcare workforce worldwide and play a key role in promoting the health of communities. Therefore, health professionals' education are more important than other fields, Because the students of these fields are the future workforce and are supposed to protect human lives as their most valuable asset. Part of this importance can be attributed to the close connection between nurses' activities and patients' health and well-being. In many definitions of nursing, emphasis has been placed not only on its scientific aspect but also on its artistic aspect (3). Nursing as an art involves creatively using knowledge and science based on skill and expertise to convey emotions and concepts to others. Using chatGPT requires interpretation, sensitivity, and active participation. Skillful use of empirical knowledge to tailor to the unique needs of patients and cautious use of creativity is essential (4). Emphasizing ethical principles is another aspect of this concept.

Among the capabilities of ChatGPT in nursing education are providing nearly instant, comprehensive, and logical textual responses to instructors' academic questions, solving university assignments, and conducting research projects. Offering quick, accurate, and convincing responses can lead students to excessively trust ChatGPT as an information source and become dependent on it. Over-reliance on artificial intelligence technologies like ChatGPT may decrease direct interactions between nursing students and instructors. The experiences of specialized instructors and experienced nurses can be invaluable and help students better understand the course material. Personal experiences usually carry a higher value than pure scientific information, and ChatGPT does not provide this added value. This issue can lead to problems in developing essential skills in nursing students. These skills include critical thinking, clinical reasoning, the ability to design a nursing care plan, and problem-solving skills (5).

Furthermore, despite the ease of access to chatbots like ChatGPT, nursing students may be less inclined to find personal solutions and engage in critical thinking. This ultimately leads to the training of nurses who may need a greater understanding of patient care situations. Communication is essential in nursing, and nursing students, by developing their communication skills, enable better patient care. Suppose nursing students are constantly engaged with digital tools and artificial intelligence. In that case, their communication abilities may decrease in real-life situations, leading to insufficient and inappropriate communication with patients in clinical settings. Technological addiction, defined as excessive use of technology, is another potential threat posed by these robots to students, which can lead to psychological, social, and physical problems (6). Therefore, creating and maintaining a balance between artificial intelligence and human capabilities in nursing education seems essential.

While the features and capabilities of these chatbots can potentially revolutionize nursing education, if not used properly, they can be a double-edged sword! Certainly, the complete replacement of artificial intelligence for human intelligence is not possible, at least in nursing. We need nurses in clinical settings who, in addition to practical skills, possess clinical reasoning abilities, analytical power, and high problem-solving skills. Artificial intelligence technologies can threaten this and be perceived as a death knell for traditional learning. Therefore, preventing their potentially dangerous threats in nursing education is recommended, especially considering regulations and guidelines before their use becomes as uncontrollable as the COVID-19 pandemic. The presence of unknown issues surrounding artificial intelligence, like the dark side of the moon, emphasizes the need for further studies on artificial intelligence threats (beyond the dark side of artificial intelligence) concurrently with the development of existing knowledge about its capabilities.

## Author contributions

ASh: Investigation, Writing – original draft, Writing – review & editing. ASa: Conceptualization, Writing – original draft, Writing – review & editing. MG: Conceptualization, Supervision, Writing – original draft, Writing – review & editing.
